# The Infrastructure Behind the Experiment: Rethinking Research Supply as a Shared Scientific Resource

**DOI:** 10.1096/fba.2026-00277

**Published:** 2026-10-26

**Authors:** Barbara A. Daily, Kimberly A. Brown, Keon W. Littrell, Alan F. Scott, Melissa V. Olson

**Affiliations:** ^1^ The Johns Hopkins University School of Medicine, Genetic Resources Core Facility Baltimore Maryland USA

**Keywords:** core facilities, research infrastructure, research operations, research supply, shared research resources, supply chain resilience

## Abstract

Modern biomedical research depends on an extensive ecosystem of shared infrastructure that enables investigators to access specialized technologies, expertise, data resources, and biospecimens. Less attention has been given to the operational infrastructure that provides reliable access to the reagents and consumables required for everyday experimental work. Although traditionally viewed as an administrative or procurement function, research supply infrastructure directly influences the cost, timeliness, continuity, and sustainability of scientific activity. This article examines centralized research supply as a form of shared scientific infrastructure through the experience of the Genetic Resources Core Facility at the Johns Hopkins University School of Medicine. What began as a small reagent‐access program evolved into an institution‐wide research supply infrastructure serving more than 5000 researchers and 846 principal investigators. In 2025, the program provided access to approximately 664,000 products and distributed more than 106,000 laboratory reagents while consolidating purchasing and distribution through a coordinated institutional framework. Over an eleven‐year evaluation period, the model also generated substantial estimated institutional economic value through negotiated pricing, reduced shipping costs, and consolidation of purchasing activity. The experience demonstrates that the value of centralized research supply extends beyond procurement efficiency. Integration within a shared‐resource environment can improve access to research materials, reduce administrative and logistical barriers, support continuity of experimental workflows, and connect purchasing decisions with scientific expertise and evolving research needs. These findings suggest that research supply can function as shared scientific infrastructure when organized around scientific access, integrated with the research ecosystem, and responsive to evolving investigator needs. Recognizing and strategically integrating this infrastructure within institutional research ecosystems provides an opportunity to strengthen scientific productivity, resilience, and long‐term sustainability.

## Graphical abstract

Centralized research supply as shared scientific infrastructure. Centralized research supply can extend beyond procurement to function as research‐enabling infrastructure when organized around four dimensions: Access, Aggregation, Integration, and Adaptability. Together, these dimensions support reliable research access, operational efficiency, resilience, and sustainability. The framework is informed by an 11‐year institutional evaluation during which more than 1.2 million research products were distributed.

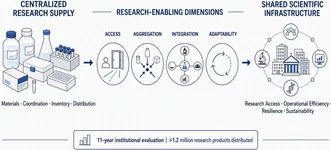

## 1 Introduction

Modern biomedical research depends on an increasingly complex ecosystem of shared infrastructure. Advanced instrumentation, computational platforms, data resources, biobanks, and scientific core facilities provide investigators with access to technologies, expertise, and resources that would be difficult or inefficient to maintain within individual laboratories [1, 2, 3, 4, 5]. These shared resources have become integral to the scientific enterprise, allowing institutions to concentrate specialized capabilities, broaden access to emerging technologies, and support increasingly multidisciplinary and data‐intensive research.

Less visible are the operational systems that allow this infrastructure—and the investigators who depend upon it—to function every day. Experimental research requires continuous access to reagents, enzymes, assay kits, laboratory plastics, cell culture materials, and other consumables that rarely appear in discussions of research infrastructure. Individually, these materials are routine; collectively, their availability is fundamental to experimental continuity. Even the most sophisticated scientific platforms cannot operate without the materials required to perform their assays, and routine experimental workflows can be delayed or interrupted when essential reagents are unavailable [6, 7]. Research supply should therefore be considered not simply as a purchasing activity, but as an issue of scientific access.

At many academic institutions, however, research supply remains highly decentralized. Individual investigators and research staff identify products, compare vendors, place and track orders, receive shipments, and maintain local inventories according to immediate experimental needs. This flexibility is essential for specialized and novel research materials, but at institutional scale it has the potential to create substantial fragmentation. Thousands of laboratories may independently purchase overlapping products from the same suppliers through separate transactions while maintaining redundant inventories and managing repeated shipments. The cumulative result can include pricing variability, duplicated administrative and logistical effort, material waste, and delays in access to experimental inputs.

The importance of reliable research supply became particularly visible during the COVID‐19 pandemic, when global shortages disrupted access to reagents, laboratory consumables, personal protective equipment, and other materials required for biomedical research and clinical activity [6, 7, 8, 9]. These disruptions demonstrated that material availability is not merely a logistical concern: when essential experimental inputs are unavailable, scientific activity can slow or stop. Research continuity therefore depends not only on funding, personnel, instrumentation, and data infrastructure, but also on systems capable of maintaining access to the physical materials required for experimentation.

Centralized research supply offers one approach to strengthening this enabling layer of the research ecosystem. Aggregating demand across investigators can support coordinated purchasing, negotiated pricing, consolidated shipments, strategic local inventory, and distribution of commonly used materials through shared infrastructure [10, 11, 12]. Similar principles have demonstrated value in healthcare and other complex organizational environments [13, 14, 15, 16]. Within academic research, however, the potential value of centralization extends beyond procurement efficiency. When research supply is integrated with core facilities and other shared scientific resources, it can connect material access with technical expertise, emerging technologies, and evolving investigator needs while reducing administrative and logistical fragmentation [17]. This perspective is increasingly relevant as research‐intensive institutions seek to sustain sophisticated scientific capabilities while controlling costs, reducing administrative burden, and strengthening operational resilience.

Importantly, centralized purchasing alone does not necessarily constitute a shared scientific resource. Many institutions maintain centralized procurement or supply functions that efficiently manage transactions, contracts, and inventory. The distinction considered here is whether research supply gains additional value when it is embedded within the shared‐resource ecosystem and organized around scientific rather than primarily administrative objectives. In this setting, material access can be connected directly with technical expertise, experimental workflows, emerging technologies, supplier relationships, and real‐time investigator demand. This integration may allow research supply infrastructure to function not simply as a mechanism for purchasing goods, but as an enabling component of the scientific enterprise.

In this article, we propose that centralized research supply infrastructure can function as a shared scientific resource when it is organized around the needs of the research community rather than transactions alone. Drawing on the experience of the Genetic Resources Core Facility at the Johns Hopkins University School of Medicine, we examine the evolution of a small reagent‐access program into institution‐wide research supply infrastructure supporting thousands of investigators. We evaluate its scale, research reach, transactional consolidation, and economic impact and use this experience to propose a conceptual framework for research‐enabling supply infrastructure centered on four dimensions: Access, Aggregation, Integration, and Adaptability.

## 2 Methods

### 2.1 Study Design and Setting

A retrospective descriptive case study was conducted to characterize the scale, utilization, transactional consolidation, and estimated economic value of a centralized research supply model within the Genetic Resources Core Facility (GRCF) at the Johns Hopkins University School of Medicine. The analysis included Core Store operational activity from January 1, 2015, through December 31, 2025. The Core Store operates as a cost‐recovery shared resource providing investigators with centralized access to research reagents, consumables, and related laboratory products.

The analysis was designed to evaluate the Core Store as an institutional research‐support infrastructure rather than individual purchasing decisions or laboratory‐level outcomes. Measures were selected to characterize longitudinal utilization, breadth of research‐community reach, consolidation of purchasing activity, and estimated institutional economic value.

### 2.2 Data Sources and Operational Measures

Operational data were obtained from internal Core Store ordering, purchasing, and financial records. Annual measures included the number of research products distributed, the value of goods distributed, and supplier purchase orders generated. Additional measures available to characterize institutional reach included researcher orders, individual customers, principal investigators served, products available through the centralized ordering platform, and participating primary institutional vendors.

Products distributed represented individual research products supplied through the Core Store during the corresponding calendar year. Supplier purchase orders represented upstream purchase orders issued by the Core Store to participating suppliers to fulfill aggregated research demand. Through the Core Store consolidation process, demand for research products was aggregated into a substantially smaller number of supplier purchase orders.

To characterize the operational scale of purchasing consolidation, a **supply consolidation ratio** was calculated as the number of products distributed divided by the number of supplier purchase orders issued during the same year. This measure represents the volume of downstream research supply activity supported per upstream supplier transaction and was used as a descriptive indicator of operational consolidation.

Research reach was characterized using available measures of researcher orders, individual customers, and principal investigators associated with Core Store activity. These measures were used descriptively to characterize the breadth and intensity of utilization across the institutional research community and were not intended to represent unique research studies or laboratories.

### 2.3 Economic Analysis

The estimated economic value associated with the centralized research supply model was evaluated across three components: negotiated discounts from supplier list prices, estimated avoided shipping costs, and modeled administrative value associated with consolidation of supplier purchasing activity.

**Pricing value** was calculated as the difference between supplier list prices and institutional prices for products distributed through the Core Store. Because supplier list price may not represent the price that every laboratory would otherwise have paid through alternative purchasing pathways, this measure was interpreted as the estimated value associated with institutional negotiated pricing rather than as directly observed cash savings.

**Avoided shipping value** was estimated using an average shipping cost of $25 for each research product distributed through the centralized infrastructure. This calculation represents shipping costs estimated to have occurred if those products had instead been obtained through decentralized laboratory‐level purchasing. Annual estimated avoided shipping value was calculated as:

Estimated avoided shipping value=Products distributed×$25

**Administrative value** was modeled by estimating the number of supplier purchase orders avoided through centralized purchasing and consolidation. For this analysis, each research product distributed was treated as a potential individual purchasing transaction under a decentralized purchasing model. The number of consolidated supplier purchase orders actually issued by the Core Store was subtracted from the number of products distributed to estimate supplier purchase orders avoided:

EstimatedPOsavoided=Products distributed−Consolidated supplierPOs

Johns Hopkins Procurement estimated the institutional administrative cost of opening, processing, and executing a purchase order through existing institutional systems at approximately $100 per PO. This estimate was applied to the modeled number of purchase orders avoided:

Estimated administrative value=EstimatedPOsavoided×$100

The $100 value represents an institutional estimate of the administrative resources required to execute a purchase order and does not include broader investigator or laboratory personnel effort associated with product selection, order initiation, tracking, receiving, problem resolution, inventory management, or other activities potentially associated with decentralized purchasing.

Total annual estimated economic value was calculated as the sum of negotiated pricing value, estimated avoided shipping value, and modeled administrative value:

Total estimated economic value=Pricing value+Estimated avoided shipping value+Estimated administrative value

Annual values were aggregated across the 2015–2025 study period to characterize cumulative estimated economic impact. Because the shipping and administrative components model costs expected to have occurred under a decentralized purchasing environment rather than directly observed reductions in institutional expenditures, the combined results were interpreted as **estimated institutional economic value rather than realized cash savings**. Financial values are reported in nominal U.S. dollars and were not adjusted for inflation.

### 2.4 Data Analysis

Analyses were descriptive. Annual operational measures were summarized using counts, monetary values, and calculated ratios, and longitudinal trends were examined across the eleven‐year observation period. No inferential statistical testing was performed because the objective was to characterize the operation and evolution of a single institutional research supply model rather than to test causal associations or compare intervention and control groups.

Changes in utilization during the COVID‐19 pandemic were examined descriptively within the longitudinal dataset. These observations were used to characterize infrastructure activity during a period of substantial research disruption and were not interpreted as evidence that centralized research supply independently caused greater institutional resilience.

## 3 Conceptual Framework and Institutional Context

### 3.1 From Procurement to Research‐Enabling Infrastructure

Centralized research supply is not inherently scientific infrastructure. Conventional procurement systems perform essential administrative functions by executing transactions, managing vendor relationships, and ensuring compliance with institutional purchasing requirements. Research supply infrastructure is distinguished by its relationship to scientific activity: its primary purpose is to ensure that investigators can reliably access the materials required to conduct research.

Drawing on the experience described here and principles common to shared research infrastructure, we propose a conceptual framework for research‐enabling supply infrastructure organized around four interrelated dimensions: Access, Aggregation, Integration, and Adaptability (Figure 1). Together, these dimensions describe how centralized research supply can extend beyond transactional procurement to function as shared scientific infrastructure.

**FIGURE 1:**
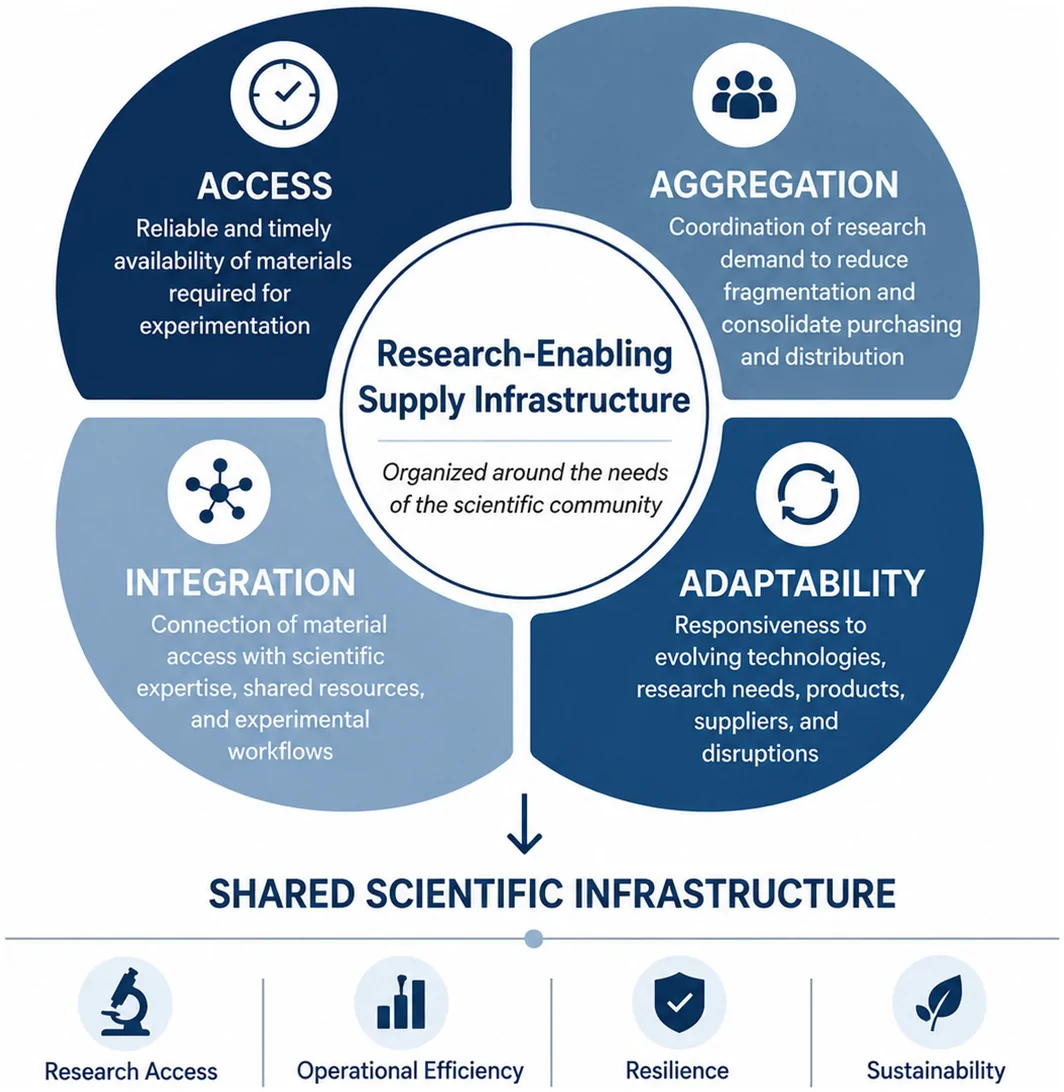
Proposed conceptual framework for research‐enabling supply infrastructure. Centralized research supply can extend beyond transactional procurement to function as shared scientific infrastructure when organized around four interrelated dimensions: Access, Aggregation, Integration, and Adaptability. Together, these dimensions emphasize reliable material availability, coordination of institutional research demand, connection to scientific expertise and shared resources, and responsiveness to evolving research needs. When organized around the needs of the scientific community, research‐enabling supply infrastructure can support broader institutional goals including research access, operational efficiency, resilience, and sustainability.

**Access**. Research supply infrastructure facilitates reliable and timely availability of materials required for experimentation. Centralized availability of commonly used reagents and consumables can reduce dependence on individual external shipments and shorten the interval between experimental need and material access. The significance of this function is scientific as well as operational: delays in obtaining essential materials can interrupt or postpone experimental workflows.

**Aggregation**. Shared supply systems coordinate demand that would otherwise remain distributed across individual laboratories. Consolidation can reduce redundant transactions and shipments, increase institutional purchasing leverage, and create visibility into patterns of research demand that are difficult to discern when activity remains fragmented across laboratories and departments.

**Integration**. Research supply provides additional value when embedded within the broader scientific support ecosystem. Connections with core facilities, biobanks, and other shared resources can link material access with technical expertise, experimental workflows, and emerging technologies. This integration allows supply capabilities to respond to scientific demand rather than functioning solely as a static purchasing or inventory system.

**Adaptability**. Research needs change continuously as new technologies emerge, priorities shift, and disruptions affect material availability. Research supply infrastructure must therefore retain sufficient flexibility to incorporate new products and suppliers, adjust inventory, and respond to evolving investigator needs without unnecessarily constraining scientific choice.

Together, these characteristics shift the objective from centralizing transactions to coordinating material access in support of research. Financial and operational efficiencies may result from this model, but they are not sufficient to define it. Research supply becomes enabling infrastructure when aggregation and operational coordination are organized around scientific access, integrated with the research environment, and responsive to changing investigator needs.

### 3.2 Research Supply Infrastructure in Practice: The GRCF Core Store

The Core Store within the Genetic Resources Core Facility (GRCF) at the Johns Hopkins University School of Medicine illustrates how research supply infrastructure can develop incrementally in response to investigator need. Rather than originating as a replacement for institutional procurement, the program developed within a scientific core facility to address a different need: timely, research‐focused access to commonly used experimental materials. Its evolution from localized reagent access to institution‐wide shared infrastructure is summarized in Figure 2.

**FIGURE 2:**
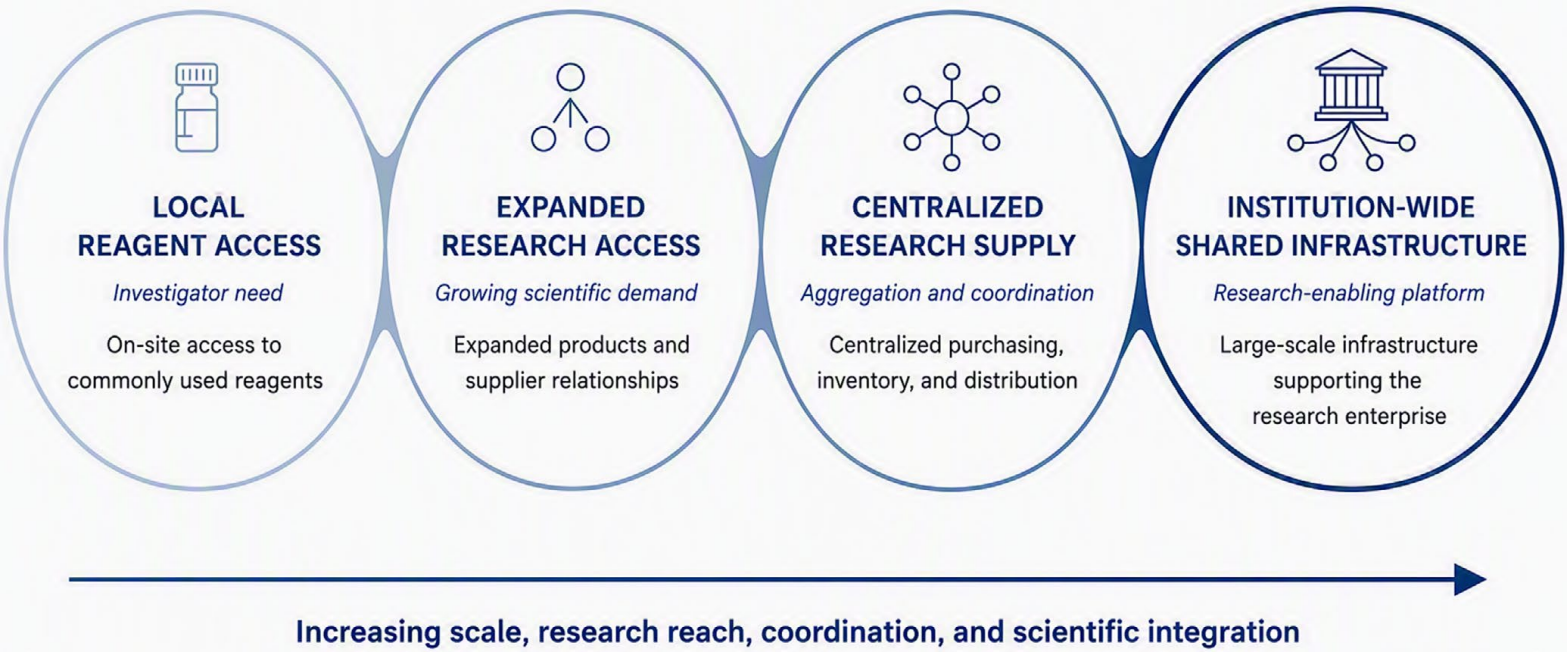
Evolution of the GRCF Core Store from local reagent access to shared institutional research infrastructure. Initially established as an on‐site reagent‐access program in response to investigator need, the Core Store expanded alongside increasing scientific demand, supplier relationships, and evolving research technologies. Over time, aggregation of purchasing, inventory, and distribution supported the transition from a localized service to an institution‐wide research supply infrastructure. The progression illustrates increasing scale, research reach, coordination, and scientific integration rather than a prescribed sequence for development of research supply infrastructure.

The program traces its origins to the 1980s, when investigators frequently experienced delays of days to weeks in obtaining routine laboratory reagents through standard purchasing channels. Shipping costs could exceed the cost of the reagents themselves, while laboratories repeatedly ordered many of the same buffers, enzymes, kits, and consumables in relatively small quantities. The GRCF responded by establishing a small on‐site “freezer program” that maintained commonly requested reagents locally, reducing the interval between experimental need and material availability.

As investigator demand increased, the program expanded. Relationships with scientific suppliers, including Bethesda Research Laboratories and New England Biolabs, broadened the range of materials available, while growth in genomics surrounding and following the Human Genome Project increased demand for molecular biology reagents and related laboratory supplies. The program evolved alongside the research community it served, expanding products, suppliers, and operational capacity as scientific needs changed.

Several features supported this evolution. Aggregation of demand allowed commonly used materials to be purchased and distributed through a coordinated infrastructure rather than repeated laboratory‐level transactions. Vendor and disciplinary neutrality allowed the program to serve investigators across the research enterprise without restricting laboratories to a single supplier or standardized set of products. Investigators also retained conventional purchasing pathways for specialized or uncommon materials.

Importantly, the Core Store remained embedded within a scientific shared‐resource environment. Its location within the GRCF connected material access with scientific services and technical expertise and created opportunities for interactions among investigators, core personnel, and suppliers. This environment also allows material access to intersect with scientific guidance, including discussions of reagent selection, compatibility with downstream applications, and adoption of products supporting emerging experimental workflows. As research technologies evolved, these interactions provided a mechanism for identifying emerging needs and incorporating new products into the supply infrastructure.

The Core Store operates under cost‐recovery principles similar to other scientific shared resources. Research activity supports the personnel, inventory, purchasing, and distribution infrastructure required to maintain the service while preserving investigator choice. Over time, this combination of scientific integration, operational coordination, and sustained utilization transformed a localized reagent‐access program into an institution‐wide research supply infrastructure. Importantly, this growth occurred through voluntary investigator adoption rather than mandated purchasing, suggesting that sustained utilization was driven by the value the infrastructure provided to the research community.

## 4 Results

### 4.1 Institutional Scale and Research Reach

The transition from localized reagent access and fragmented laboratory‐level purchasing to centralized research supply infrastructure is reflected in the scale and persistence of Core Store utilization. From 2015 through 2025, the program distributed more than 1.2 million research products across the Johns Hopkins research enterprise, with annual distribution generally exceeding 100,000 products, demonstrating sustained use across changing scientific and operational conditions.

This activity was supported through a substantially smaller number of upstream supplier transactions. Across the study period, approximately 2600 to 3500 supplier purchase orders annually supported distribution to the research community. In 2025, 106,684 products were distributed through 2624 supplier purchase orders, corresponding to 40.7 products distributed per supplier transaction—the highest consolidation ratio observed during the eleven‐year period (Figure 3).

**FIGURE 3:**
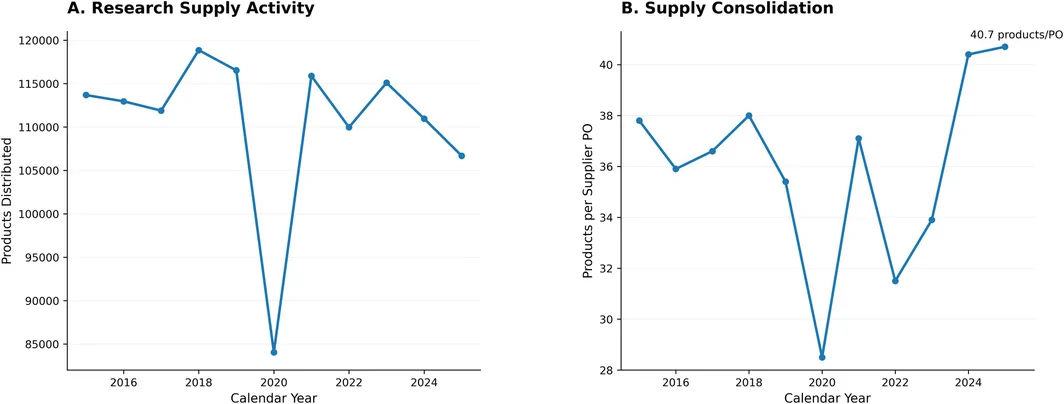
Longitudinal scale and purchasing consolidation of the GRCF Core Store, 2015–2025. (A) Annual number of research products distributed through the centralized research supply infrastructure. (B) Supply consolidation ratio, calculated as the number of products distributed divided by the number of supplier purchase orders issued during the corresponding year. The ratio provides a descriptive measure of the volume of downstream research supply activity supported per upstream supplier transaction. The decline in product distribution observed in 2020 occurred during the COVID‐19 disruption to research activity.

The breadth of the infrastructure is similarly reflected in the community it serves. In 2025, the Core Store processed 40,341 researcher orders from 5083 customers representing 846 principal investigators. The infrastructure provided access to 663,987 products from 13 primary institutional vendors and distributed approximately $18.7 million in research materials (Table 1).

**TABLE 1:** Operational scale and Research Reach of the GRCF Core Store, 2025. Current‐year operational characteristics of the centralized research supply infrastructure, including the value and volume of materials distributed, researcher ordering activity, breadth of institutional utilization, and product and vendor availability. Principal investigators and individual customers represent users associated with Core Store activity during the 2025 calendar year.

Metric	2025 value
Value of goods distributed	$18.7 M
Researcher orders processed	40,341
Principal investigators served	846
Individual customers served	5083
Research products distributed	106,684
Products available	663,987
Participating vendors	13
Average order value	$465

Longitudinal activity also illustrates operation through a period of substantial disruption. Product distribution declined from 116,530 items in 2019 to 84,045 in 2020 during the COVID‐19 pandemic and rebounded to 115,882 in 2021. Although these observational data cannot establish that centralization itself improved resilience, they demonstrate that the infrastructure remained operational during the disruption and accommodated the subsequent recovery in research activity.

Together, these data demonstrate sustained utilization of a shared supply infrastructure at institutional scale and substantial consolidation of laboratory demand into coordinated upstream purchasing activity.

### 4.2 Institutional Value: Access, Efficiency, and Sustainability

The value of research supply infrastructure is not captured by transaction volume alone. A coordinated infrastructure can create value at multiple levels by facilitating investigator access, shifting repetitive administrative and logistical activities away from individual laboratories, aggregating institutional demand, and generating efficiencies that contribute to long‐term sustainability.

For investigators, the most immediate benefit is simplified access to commonly used research materials. Local inventory and institutional distribution can reduce dependence on individual external shipments and the need for laboratories to maintain extensive inventories of routine supplies. Centralized infrastructure can also consolidate portions of the ordering, tracking, receiving, and distribution activity that would otherwise be repeated across individual laboratories, allowing scientific personnel to interact with a common access platform.

At the institutional level, aggregation reduces duplication across purchasing, receiving, invoicing, and distribution while increasing purchasing leverage. The degree of consolidation observed over the study period demonstrates how a large volume of researcher demand can be supported through substantially fewer supplier transactions. This structure also enables negotiated institutional pricing and consolidation of shipping activity.

The economic value associated with this model was estimated using three components: discounts from supplier list prices, avoided shipping costs, and modeled reductions in purchase‐order processing (Table S1). Between 2015 and 2025, annual discounts from list price ranged from approximately $2.6 million to $4.1 million, while estimated avoided shipping costs ranged from approximately $2.1 million to $3.0 million. In 2025, these components totaled approximately $6.61 million.

Administrative value was modeled by comparing the number of products distributed with the number of supplier purchase orders required by the centralized system and applying an estimated processing cost of $100 per purchase order avoided. Using this approach, modeled administrative value ranged from approximately $8.1 million to $11.6 million annually. Across the eleven‐year period, the combined estimated economic impact of negotiated discounts, avoided shipping, and modeled transaction consolidation was approximately $186.7 million. Because portions of this calculation depend on assumptions rather than directly observed expenditures, this figure is best interpreted as an estimate of institutional economic value rather than realized cash savings (Table S1).

These efficiencies also support the infrastructure itself. The Core Store's cost‐recovery model allows utilization to support the personnel and operations required to maintain access, while aggregation generates efficiencies for investigators and the institution. Financial performance is therefore not the ultimate purpose of the model, but one mechanism through which reliable research access can be sustained over time.

## 5 Discussion

This case demonstrates how operational systems developed around routine scientific needs can acquire the characteristics of shared research infrastructure when they become broadly utilized, scientifically integrated, and operationally durable. Viewed through the proposed Access–Aggregation–Integration–Adaptability framework, the significance of the Core Store model lies not simply in centralizing purchasing, but in organizing material access around the needs of a large and evolving research community.

### 5.1 Integration With the Scientific Ecosystem

A central finding from this experience is that centralization alone does not transform procurement into research infrastructure. Institutional procurement offices provide essential expertise in contracting, purchasing compliance, financial controls, and vendor management. The value of locating research supply within the shared‐resource ecosystem is therefore not that it replaces these institutional functions, but that it adds a scientific interface between procurement capabilities and the research community.

The Core Store developed alongside scientific core services rather than as an isolated administrative purchasing function. As a result, material access occurs within an environment connected to investigators, technical expertise, experimental workflows, emerging technologies, and suppliers. Core personnel are positioned close to the scientific activities that generate demand and can recognize changes in reagent requirements as technologies and workflows evolve. Interactions among investigators, shared‐resource personnel, and suppliers can also inform product selection, identify compatibility with downstream applications, and facilitate the introduction of materials supporting new experimental approaches.

This positioning creates a bidirectional interface between scientific demand and institutional supply capability. Investigator and core‐facility needs inform product availability and inventory, while suppliers and scientific staff can introduce new materials, technologies, and applications through established shared‐resource channels. Aggregated utilization can further provide visibility into changing research demand that may be less apparent when purchasing activity is distributed across individual laboratories.

Thus, the added value of a shared‐resource‐based model lies not simply in where the supply function is administratively housed, but in its proximity and responsiveness to the scientific community. Conventional institutional procurement remains essential for executing transactions and managing institutional purchasing requirements; research supply infrastructure complements these functions by translating scientific demand into coordinated material access. The objective becomes not simply maintaining inventory or reducing transaction costs, but maintaining an access infrastructure responsive to the experimental environment it supports.

### 5.2 Strategic Centralization While Preserving Investigator Choice

Academic research requires flexibility, particularly for specialized reagents, emerging technologies, and unconventional experimental approaches. A centralized model that attempts to standardize all research purchasing could therefore create barriers rather than remove them.

The Core Store instead functions as a complementary pathway. Centralization is applied where aggregation creates value, particularly for recurring or broadly utilized materials, while investigators retain conventional purchasing options for specialized needs. Vendor neutrality further supports this approach by allowing product offerings to respond to research demand rather than dependence on a single supplier. The relevant goal is therefore not maximal centralization, but strategic coordination where coordination improves access or efficiency without unnecessarily restricting scientific choice.

### 5.3 Scale Creates Opportunities Beyond Purchasing Efficiency

Institutional scale creates value beyond negotiated pricing. Consolidated activity provides visibility into patterns of material utilization, emerging demand, and changes in research activity that are difficult to identify in fragmented laboratory‐level systems [16]. As research‐support operations become increasingly data driven, these longitudinal data may support more sophisticated inventory management, demand forecasting, supplier evaluation, and identification of emerging technology adoption.

Such visibility may also have implications for research resilience. Institutions with insight into aggregate demand and relationships across multiple suppliers may be better positioned to identify shortages, locate alternatives, and coordinate responses to disruption [15, 16]. Shipment consolidation may additionally reduce the packaging and transportation associated with repeated laboratory‐level deliveries, although environmental impacts were not directly measured in this study and represent another opportunity for future evaluation. These potential operational, resilience, and environmental benefits warrant direct investigation.

### 5.4 Sustainability and Transferability

Long‐term research infrastructure must create sufficient value to sustain participation by the communities that use and support it. In this model, investigators receive convenient access and institutional pricing; the institution benefits from aggregation and reduced transactional complexity; and suppliers gain a coordinated mechanism for serving a large research community. This alignment may help explain how the program expanded over several decades without requiring mandatory adoption. The potential transferability of centralized research supply models has also been recognized within the broader shared research resource community. FASEB has previously identified the Johns Hopkins Core Store as a model that could be broadly adopted, highlighting its potential to reduce investigator time and packaging associated with research supply [18].

The model will not translate identically to every institution. The scale and concentration of the Johns Hopkins biomedical research enterprise, existing shared‐resource infrastructure, purchasing volume, and long‐standing supplier relationships contributed to its development. Smaller, less research‐intensive, or geographically distributed institutions may require different inventory, distribution, staffing, and financial approaches. The transferable principle is therefore not the specific structure of the Core Store, but the organization of research supply around access, aggregation, scientific integration, adaptability, and sustainable operation.

### 5.5 Limitations and Future Directions

Several limitations should be considered when interpreting these findings. The economic analysis includes assumptions that do not represent directly observed reductions in institutional expenditures. Discounts are calculated relative to supplier list prices and may not represent the price that every laboratory would otherwise have paid. Shipping savings assume an avoided shipping cost of $25 per product distributed, and administrative savings are modeled using an assumed $100 processing cost for each avoided purchase order. These estimates should therefore be interpreted as measures of potential institutional economic value rather than audited cash savings.

The present analysis also does not directly measure several outcomes hypothesized to result from centralized research supply, including reductions in investigator administrative time, experimental delays, laboratory inventory, product expiration and waste, environmental impact, or improvements in research continuity. Future studies could directly evaluate these outcomes through time‐motion analyses, delivery‐time comparisons, inventory and waste metrics, environmental assessment of shipment consolidation, investigator experience measures, and analysis of research interruptions associated with material availability.

More broadly, future work could examine whether research supply data themselves provide useful measures of institutional research activity. Patterns of material utilization, technology adoption, and changing demand may offer an additional source of operational intelligence for planning shared resources and anticipating emerging scientific needs.

## 6 Conclusion

Modern experimental science depends not only on visible infrastructure such as major instrumentation, specialized facilities, and computational resources, but also on reliable access to the materials required to use them. Research supply represents one of these less visible enabling systems.

The experience of the GRCF Core Store demonstrates that centralized research supply can become shared scientific infrastructure when it is organized around scientific access, integrated with the broader research ecosystem, responsive to evolving investigator needs, and sustained at institutional scale. Its distinguishing value is not centralization alone, but the integration of material access with the scientific expertise, workflows, technologies, and evolving demand of the research community. Recognizing these systems as part of the research infrastructure landscape expands how institutions think about the resources required to support productive and sustainable science.

## Author Contributions

B.A. Daily, A.F. Scott and M.V. Olson conceived and designed the research; B.A. Daily, K.A. Brown and K.W. Littrell performed the research and acquired the data; K.A. Brown and M.V. Olson analyzed and interpreted the data. M.V. Olson drafted the manuscript and all authors were involved in revising the manuscript.

## Conflicts of Interest

The authors declare no conflicts of interest.

## Supporting information


**Table S1:** Estimated Economic Value Associated with the GRCF Core Store Centralized Research Supply Model, 2015–2025.

## Data Availability

Stored in repository.
