# Supplementary material for: The Infrastructure Behind the Experiment: Rethinking Research Supply as a Shared Scientific Resource
Source: FASEB Bioadv. 2026 Oct 26;8(11):e70159. doi: 10.1096/fba.2026-00277 (PMC13702830; doi:10.1096/fba.2026-00277)
Supplement: Supplementary file 1 [file FBA2-8-e70159-s001.docx]

**Supplemental Table 1.** Estimated Economic Value Associated with the GRCF Core Store Centralized Research Supply Model, 2015–2025.

***Note:*** *Direct economic value includes estimated avoided shipping value, calculated at $25 per product distributed, and pricing value associated with discounts from supplier list prices. Administrative value was modeled by treating each product distributed as a potential individual purchasing transaction under a decentralized purchasing model and subtracting the number of consolidated supplier purchase orders actually issued. An institutional processing cost of $100 per purchase order, based on an estimate provided by Johns Hopkins Procurement, was applied to estimated purchase orders avoided. Values represent modeled institutional economic value and should not be interpreted as directly observed reductions in institutional expenditures.*
